# Recognizing flu-like symptoms from videos

**DOI:** 10.1186/1471-2105-15-300

**Published:** 2014-09-12

**Authors:** Tuan Hue Thi, Li Wang, Ning Ye, Jian Zhang, Sebastian Maurer-Stroh, Li Cheng

**Affiliations:** Canon Information Systems Research Australia, Sydney, Australia; Department of ECE, Northeastern University, Boston, USA; Bioinformatics Institute, A*STAR, Singapore, Singapore; Advanced Analytics Institute, FEIT, University of Technology Sydney, Sydney, Australia; School of Biological Sciences, Nanyang Technological University, Singapore, Singapore; National Public Health Laboratory, Communicable Diseases Division, Ministry of Health, Singapore, Singapore; School of Computing, National University of Singapore, Singapore, Singapore

## Abstract

**Background:**

Vision-based surveillance and monitoring is a potential alternative for early detection of respiratory disease outbreaks in urban areas complementing molecular diagnostics and hospital and doctor visit-based alert systems. Visible actions representing typical flu-like symptoms include sneeze and cough that are associated with changing patterns of hand to head distances, among others. The technical difficulties lie in the high complexity and large variation of those actions as well as numerous similar background actions such as scratching head, cell phone use, eating, drinking and so on.

**Results:**

In this paper, we make a first attempt at the challenging problem of recognizing flu-like symptoms from videos. Since there was no related dataset available, we created a new public health dataset for action recognition that includes two major flu-like symptom related actions (sneeze and cough) and a number of background actions. We also developed a suitable novel algorithm by introducing two types of Action Matching Kernels, where both types aim to integrate two aspects of local features, namely the space-time layout and the Bag-of-Words representations. In particular, we show that the Pyramid Match Kernel and Spatial Pyramid Matching are both special cases of our proposed kernels. Besides experimenting on standard testbed, the proposed algorithm is evaluated also on the new sneeze and cough set. Empirically, we observe that our approach achieves competitive performance compared to the state-of-the-arts, while recognition on the new public health dataset is shown to be a non-trivial task even with simple single person unobstructed view.

**Conclusions:**

Our sneeze and cough video dataset and newly developed action recognition algorithm is the first of its kind and aims to kick-start the field of action recognition of flu-like symptoms from videos. It will be challenging but necessary in future developments to consider more complex real-life scenario of detecting these actions simultaneously from multiple persons in possibly crowded environments.

## Background

While the recent swine flu pandemic was luckily less severe than initially thought, there remains a constant threat of mutated or reassorted influenza strains that give rise to new outbreaks that could range from small local clusters [1] to seasonal epidemics [2] or even global pandemics [3, 4]. Similarly, history has also shown us that previously unknown pathogens such as the SARS coronavirus could emerge and cause serious outbreaks [5]. Just in 2012 and 2013, there were 2 new outbreaks of different viruses with pandemic potential, MERS-CoV [6] and H7N9 [7], triggering increased surveillance alerts. Respiratory diseases often manifest themselves through similar flu-like symptoms and early detection of new outbreaks is of central importance in order to delay or prevent their escalation and wider spread. However, classical surveillance systems are mostly relying on time-delayed and costly virological tests requiring hospital or physician visits [8–10].

One potential alternative is to detect typical flu-like symptom in human behaviors, by automatically analyzing video footage from public areas such as airports, bus stations, which exploits the existing vision-based surveillance infrastructure in public venues. This will provide a unique valuable source of information that is complementary to the existing public health monitoring network. Under this context, we make a first attempt on the recognition of typical flu-like symptoms: sneeze and cough actions, and propose a novel discriminative approach which is further evaluated on a new Sneeze-Cough action dataset.

**Major contributions** Our first contribution is a new video action dataset^1^ dedicated towards the problem of flu-like symptoms detection that is of central importance in early surveillance of respiratory disease outbreaks. A series of experiments are conducted with performance analysis that reveals some of the characteristics of this dataset. Our second contribution is two novel types of Action Matching Kernels (AMKs) that are shown to perform competitively comparing to the state-of-the-art methods. In particular, we show that Pyramid Match Kernel [11] and Spatial Pyramid Matching [12] are both special cases of the proposed kernels. The kernels are also closely connected to the recent developments in Hough transform [13, 14].

**Related work** Current respiratory disease surveillance systems are known to lag significantly behind the onset of outbreaks [15, 16], mostly due to their heavy reliance on virological and clinical data including physician visits. Very recently a web-based surveillance tool has been developed by Google [17], which is made possible through search engines by taking advantage of the social health-seeking behavior of patients. There are nonetheless concerns that there sometimes exists non-negligible bias in the detection results driven by disease publicity rather than the disease itself. The work presented in this paper, to our best knowledge, is the first to examine this problem with the help of vision-based surveillance and analysis.

Research on video action recognition and retrieval [18] has recently witnessed a dramatic increase, mainly due to the vast demand to analyze and understand human actions from video footage of everyday life, and from web hosts such as YouTube, MySpace Videos, Flickr, and ScienceStage. Established methods for modeling and analyzing human actions are often generative statistical approaches, especially the Markov models *e.g.* [19, 20]. Recently, the discriminative learning scheme has also been extended to allow structured predictions, *e.g.* Conditional Random Fields [21]. They nevertheless often rely on learning with sophisticated parametric models.

Similar to a number of recent works [22–25], we also assume a human action can be sufficiently described by a set of local features in space-time. A local feature typically comes with two aspects: a descriptor vector and its space-time location. As the number and locations of the local features are usually not fixed, often a bag-of-words (BoW) method is utilized to map the feature descriptors to a histogram vector in the space spanned by codewords, as in [11, 24, 26], or hierarchical codewords as described in the Pyramid Match Kernel [11]. The BoW representation has demonstrated impressive performance on image and action analysis tasks. Nevertheless it does not retain information regarding space-time layout of the local features.

On the other hand, the spatial (or space-time) layout of local features has long been regarded as an important cue to infer the existence of a global object from local features. The elegant Hough transform [27] is originally devised to detect lines and circles. An important generalization is developed by Ballard [28] to detect objects of arbitrary shapes. Leibe *et al.* in their seminal work [13] consider a probabilistic variant of the Hough transform, where the BoW model is integrated into the voting space by means of conditional and posteriori probabilities. This is further followed by [14] where a dedicated max-margin learning method is developed. Throughout these methods, a crucial step is the construction of a voting space, where all local features are made to vote for the existence and if so, the location of the global object they belong to. An interesting observation is that this voting space is employed by [14] in an implicit manner. As clearly revealed from Equations (12) and (13) of [14], the model or the parameter vector *W* is *implicitly* related to the voting space: *W* is interpreted as weights for the activations of codewords, where influence from the voting space is implicitly carried out via the activations. A latent variant has also been used for object detection [29].

Recently there have been attempts to integrate the two sources of information: BoW and the space-time layout. The Spatial Pyramid Matching [12], a probabilistic variant of Hough transform (also called Implicit Shape Model) [13], and utilizing the skeleton structure of human body [30], are such examples. In the next section, we show that our AMK *explicitly* incorporates the space-time layout and the BoW model. We will also show that the Pyramid Match Kernel and the Spatial Pyramid Matching are *special cases* of our proposed AMKs with proper feature extensions. Section ‘Sneeze-Cough: a public health surveillance dataset’ will describe our new Sneeze-Cough dataset in details, and followed the experimental results in Section ‘Results and discussion’.

## Methods

**Figure 1:**
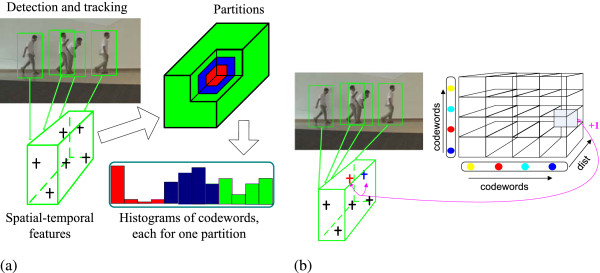
**The unary and binary feature extensions.** An illustration of the unary and binary feature extensions for the proposed Type I Kernels. Here local features (‘+’s) of an action is contained in a space-time volume displayed as a green cube. Besides residing in this local coordinate, each feature also has a descriptor that is further mapped to the histogram of codewords representation using the bag-of-words model. This figure displays two examples of the proposed Type I Kernels. **(a)** presents a unary extension where the local volume is further partitioned into concentric layers. **(b)** demonstrates a binary extension scheme that considers the distance and codeword entries of feature pairs. Note other extension schemes are also possible.

In this section, we propose two types of Action Matching Kernels (AMKs) that integrate both BoW and space-time layout. The first type is by means of unary and binary extensions, while the second type is a modification of the successful Pyramid Match Kernel. As shown in Figure 1, an action can be naturally depicted by a set of local features within a space-time action volume (the green cube illustrated in Figure 1(a) bottom left), which we also refer to as action volume or simply volume when there is no confusion. In both cases, an input action corresponds to both a BoW model consisting of *K* codewords, as well as the features’ locations in the space-time volume.

### AMK Type I: unary and binary extensions

Presented with such an action, below we describe a set of kernel design recipes that are able to integrate both the BoW representation and the space-time locations of local features.

**Unary extensions** A unary extension partitions the volume into disjoint parts. One such scheme is to partition into concentric layers, as displayed in Figure 1(a). By pooling the codeword assignments of these features in their BoW representation, one partition is characterized by a histogram of length *K* when *K* codewords are used. A length *K*×*S* vector is thus produced as a unary extension, by concatenating over *S* partitions.

Other partition schemes are also possible. For example, partitioning the volume into half in each dimension results in 2×2×2 blocks, and is denoted as *S*=8. We can further partition each block into smaller ones, where each block has its own histogram. Interestingly, this is the same three-layer spatial pyramid as depicted in Figure 1 of Spatial Pyramid Matching [12]. The only difference is that here we consider a 3D space-time volume instead of a 2D image space. By summing all the histograms over layers with proper weights, and by using histogram intersection similarity measure, we get back exactly the Spatial Pyramid Matching [12].

In fact, the degenerate case of unary extensions by setting *S* to 1 returns the original BoW model. Meanwhile, by fixing *S* to 1, considering a BoW model with hierarchical codewords, and by using histogram intersection similarity measure, the Pyramid Match Kernel [11] is recovered.

**Binary extension** Different from the unary extensions, a binary extension considers the interactions between a pair of features in space-time. Figure 1(b) provides such an example, where similar to the concept of co-occurrence matrix, a 3-dimensional array or 3-tensor is used to accumulate the counts of feature pairs using both volume and BoW representations, indexed by (codewords, codewords, distance). Naively this leads to a vector of length *K*×*K*×*S*, by accumulating the quantized distance of each feature pair with *S* possible outcomes. In practice it is further summarized into a more compact vector representation: For a fixed distance, (a) a *K*-dim vector is extracted from the diagonal elements. (b) a *K*-dim vector is obtained by summing over all the off-diagonal elements row-wise. For both cases the output vectors are normalized to sum to 1. As each case ends up giving a *K*×*S* vector, a concatenation of both finally leads to a vector representation of length 2*K*×*S*.

**From feature extensions to kernels** It is straightforward to carry on and build a kernel from the extended vectors mentioned above. In fact, a kernel can be built by considering different feature extension, by examining on a variety of similarity measures (*e.g.* linear, *χ*^2^, histogram intersection, radial basis function), and by choosing from hierarchical vs. standard codeword representations. A family of kernels can thus be devised using the above recipes, where the examples we illustrate in the paper comprise only a small fraction.

### AMK Type II: a modified pyramid match kernel

**Original pyramid match kernels** In the original Pyramid match kernel paper [11], a video action is represented as a set of local features descriptors excluding their space-time location information. Therefore, an action of interest is represented as . This is followed by building hierarchical codewords using *e.g.* hierarchical K-means. In each scale *l*∈0,…,*L*-1, a histogram *H*_*l*_ of codewords can be computed. Note the length of corresponding histogram decreases as we navigate to the upper layers of the hierarchy. By concatenating these histograms, the action *P* is then characterized as a feature vector *Ψ*(*P*) = (*H*_0_(*P*),…,*H*_*L*-1_(*P*)). As in [11], The kernel function between two actions *P* and *Q* is thus defined by
1

Here *w*_*l*_ is used to limit the contribution from a particular scale of histogram, as inversely proportional to its scale, *w*_*l*_ = 2^-*l*^. *N*_*l*_ is the partial increment from level *l*-1 to level *l*,
2

*τ* denotes the histogram intersection:
3

which can be equivalently written as
4

where *ID*(·) stores the codeword index of a local feature, *δ* is the indicator function, and *k* is an index of the set of codewords.

**Figure 2:**
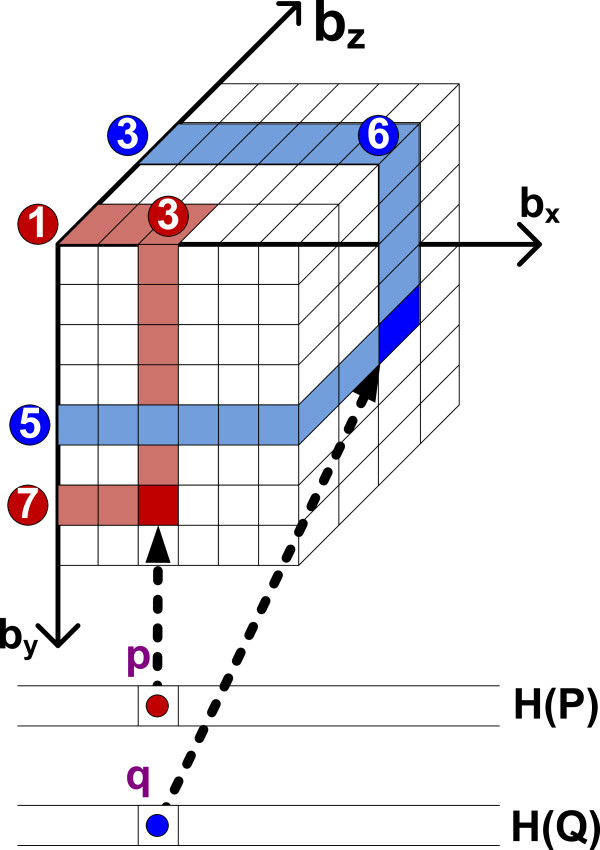
**A toy example.** A toy example to illustrate the geometric measure *M*_*l*_(*p*,*q*) of Eq. (5) for a feature pair (*p*,*q*) at codeword *k*, and with *H* ^*k*^(*P*) = *H* ^*k*^(*Q*) = 1. Pyramid Match Kernel [11] returns 1, since it ignores feature space-time locations; Our AMK Type II returns a matching score using Eq. (6) as: , which indicates the geometric affinity of the two features.

**AMK type II** As illustrated in Figure 2, instead of using histogram intersection of Eq. (3), we consider a matching function by modifying Eq. (4) to incorporate space-time locations of local features:
5

where *M*_*l*_(*p*,*q*) is a geometric measure of the feature pair and is computed as their affinity in the space-time volume,
6

As shown in Figure 2, (, , ) refers to its quantized 3D location in the volume, while *D*_*x*_, *D*_*y*_, and *D*_*t*_ denote the number of quantization levels on each of the dimensions, respectively. It is easy to check that for the trivial case *D*_*x*_ = *D*_*y*_ = *D*_*t*_ = 1, Eq. (3) is recovered from Eq. (5). In other words, the Pyramid Match Kernel [11] can be regarded as *a special case* of AMK Type II when no spatial and temporal constraints are enforced.

### Mercer kernels and the action volume

**Action matching kernels are Mercer kernels** It is easy to check that AMKs Type I are Mercer kernels (i.e. the kernel matrix is positive semi-definite (p.s.d.) [31, 32]) as long as proper similarity measures such as *χ*^2^, and histogram intersection are utilized. An important property of AMK Type II as defined by Eqs. (1), (2), (5) and (6) is that it is a Mercer kernel. This is clear from the fact that Eq. (6) is a p.s.d, as well as the fact that Mercer kernels are closed under positive scaling and summation operations [31] and the weights *w*_*l*_ are always positive. Endowed with a Mercer kernel, the induced convex optimization problem is guaranteed to produce a unique optimal solution using the support vector machine (SVM) classifiers [31]. In practice, we use the binary/multiclass algorithms of LibSVM [33] with customized kernels.

**The action volume** An action is naturally bounded in 3D space-time, as *e.g.* illustrated in Figure 1(a) bottom left. In fact this is a property inherit in the problems regarding action recognition and retrieval. In a typical recognition dataset such as KTH [22], where there is only one person performing an action in a video, the action is bounded by the size of the frames. One possible scheme is to consider a local coordinate with its origin fixed to the center of these features, and to explicitly examine all possible scales in a manner similarly to that of the Hough voting space. A simple scheme is instead considered in this paper, where the action volume is determined by aligning the bounding boxes detected using a human detector [34]. As a result, its scale is also implicitly decided.

### Sneeze-Cough: a public health surveillance dataset

**Figure 3:**
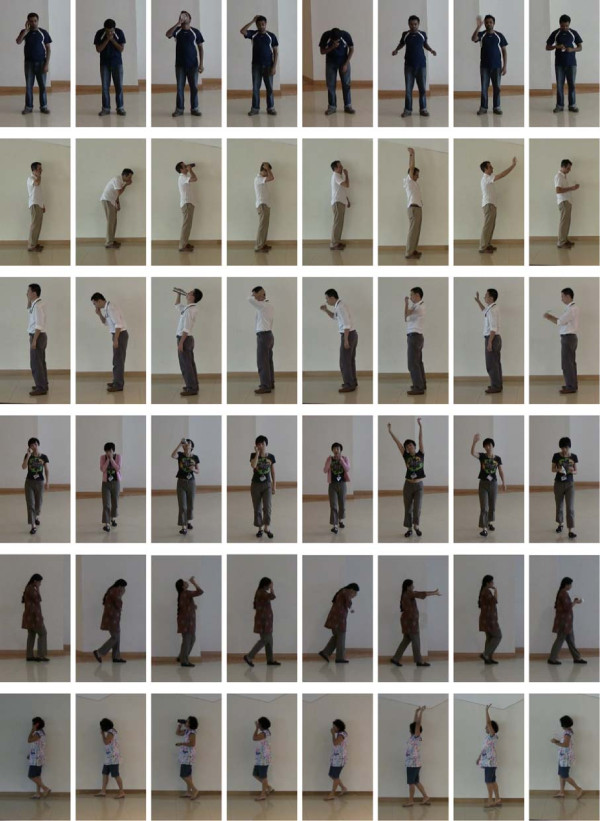
**Snapshots of Sneeze-Cough action recognition videos.** Snapshots of Sneeze-Cough action recognition videos. From left to right shows *eight* actions: answer phone call, cough, drink, scratch face, sneeze, stretch arm, wave hand and wipe glasses. From top to bottom shows *six* pose-and-view variations: stand-front, stand-left, stand-right, walk-front, walk-left, and walk-right.

We describe here the new Sneeze-Cough video dataset that tailors to the specific challenges and characteristics of recognizing flu-like behavior symptoms in public areas. Note written consent on publication and use of the video data was obtained from each volunteer and the study was cleared by the Bioinformatics Institute ethics committee represented by the executive director. This dataset contains 960 color video clips of imitated surveillance video settings, collected from 20 human subjects (8 females and 12 males) of 20 to 50 years old using a Canon VIXIA HF20 camcorder. A gallery of sample frames are displayed in Figure 3. The data acquisition process is carried out in an indoor environment with semi-controlled lighting condition (sun lights through windows are present in some of the videos), and the camera is mounted on a tripod mimicking the relative height of a typical surveillance camera. Each clip contains one specific action performed by one subject in a particular view and pose. Video shots are normalized at 480 × 290 resolution, with stream rate of 5 frame per second, each lasts for around 15 seconds. In addition to the two flu-like behaviors, namely *sneeze* and *cough*, six common background action types are also included: *drinking*, *phone calling*, *scratching head*, *stretching arms*, *wiping glasses* and *waving hands*. Note we deliberately cover a spectrum of possible background action types that are relatively close to our actions of interest. In addition, each human subject performs each action six times under 2 different poses (standing and walking) and 3 different views (roughly frontal/left/right). We also perform horizontal flip on each video to produce an additional video set of reflective views, which results in a final set of 1920 videos.

## Results and discussion

Throughout the experiments, the following parameters are used: For AMK Type I and II, the number of codewords is fixed to *K* = 1024. By default, *χ*^2^ similarity measure is used for AMK Type I; Meanwhile AMK Type II employs histogram intersection, together with a hierarchical codewords of 4 levels. These two different similarity measures are utilized here to showcase the flexibility of incorporating various similarity measures into the proposed AMKs. LibSVM [33] is used with the trade-off parameter *C* = 10. To verify that the proposed AMKs are able to accommodate different local features, two publicly available local feature detectors & descriptors are considered, namely HOGHOF (also called Space Time Interest Point) [22], and cuboid [23].

**Accuracy measures** For KTH we use the standard accuracy measure by averaging the diagonal values from the row-normalized confusion matrix. For binary classification, this becomes ^2^, which however is problematic for datasets with imbalanced class distributions such as Sneeze-Cough: As 3/4 of the Sneeze-Cough examples belongs to background actions category, and using the standard accuracy measure, a rate of 75*%* is reached when a classifier is biased towards blindly assigning every example to background actions. This leads to the utilization of precision and recall, which are computed by  and , respectively. We thus adopt a *different* accuracy measure of  for this binary classification task, which can be regarded as a lower-bounding summary of the (precision, recall) pair.

**KTH** The KTH dataset [22] contains 2391 video shots from 25 actors repeatedly performing 6 different action types under 4 different background contexts. The actions include boxing, handclapping, handwaving, jogging, running, and walking. To facilitate direct comparison, the same split scheme of [22] is adopted in our experiments, where videos from 16 persons are used for training, and the other 9 persons are retained for testing. Table 1 compares results of our proposed AMKs with reported works. Our implementation of the two baseline methods (88.2*%* for cuboid and 88.5*%* for HOGHOF) is consistent with what has been reported in the literature (89.1*%* [35] for cuboid and 91.8*%* [35] for HOGHOF). And the results of the proposed AMK methods (with best rate of 93.4*%* with cuboid and 94.2*%* with HOGHOF) are competitive when comparing to the state-of-the-art approaches.

**Table 1:** **Comparisons of recognition accuracies on KTH dataset**

Method	Brief description	Acc. %
ICPR’04 [22]	HOGHOF + SVM	71.7
CVPR’07 [36]	cuboid + WX-SVM	91.6
BMVC’09 [35]	cuboid + BoW+ *χ* ^2^	89.1
	HOGHOF + BoW + *χ* ^2^	91.8
ECCV’10 [37]	convolutional nets	90.0
In this paper	cuboid + BoW + *χ* ^2^ (baseline)	88.2
	cuboid + AMK I *U*_*c*_ S=2	91.7
	cuboid + AMK I *U*_*b*_ S=8	93.0
	cuboid + AMK I B S=7	92.7
	cuboid + AMK II	93.2
	HOGHOF + BoW + *χ* ^2^ (baseline)	88.5
	HOGHOF + AMK I *U*_*c*_ S=3	91.1
	HOGHOF + AMK I *U*_*b*_ S=27	95.2
	HOGHOF + AMK I B S=5	92.3
	HOGHOF + AMK II	93.5

Comparisons of recognition accuracies on KTH dataset. Here shorthands of AMK I and II are used for AMK type I and type II kernels, respectively. *U*_*c*_ (*U*_*b*_) refers to the concentric (block) partition scheme in unary extension. B is for binary extension.

**Sneeze-Cough** For the Sneeze-Cough dataset, we use 15 persons for training, and retain the rest 5 persons for testing. We would like to emphasize that this dataset is significantly different and is more challenging comparing to the KTH dataset. First, the actions in this dataset, except for hand-waving, are usually of short time-span, in contrast to actions such as walk or boxing that usually consist of a good number of repetitive action cycles. Second, there exist large variations within the sneeze and cough actions over *e.g.* different genders, ages, and views. This is further complicated by the fact that the background actions commonly seen in public areas (such as phone calling, scratching head) are often very similar in appearance to flu-like symptoms. Meanwhile these 6 background actions by themselves are highly complex and exhibit large variations as well, as indicated in the sample frames of Figure 3.

**Figure 4:**
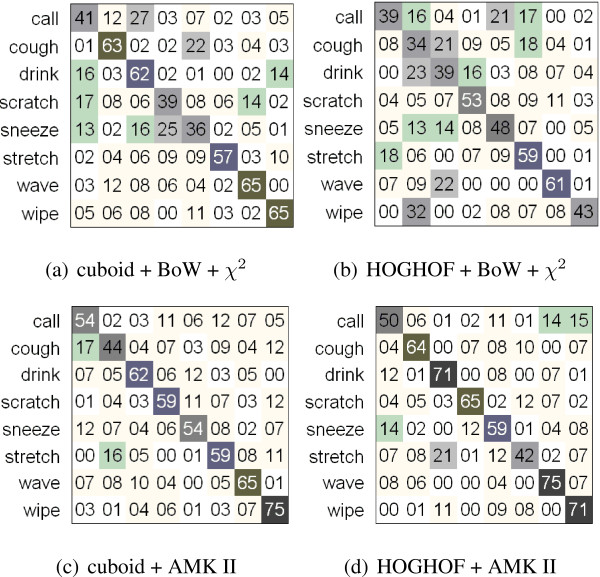
**Confusion matrices of Sneeze-Cough 8-class recognition.** Confusion matrices of Sneeze-Cough 8-class recognition.

**Figure 5:**
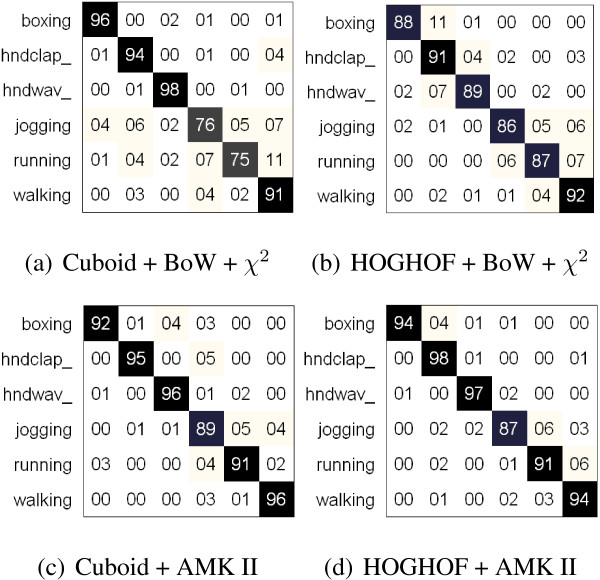
**Confusion matrices of KTH 6-class recognition.** Confusion matrices of KTH 6-class recognition.

By experimenting with 8-class recognition tasks, confusion matrices are obtained to facilitate our investigation into the inter- and cross- actions pattern of this new dataset. Figure 4 presents the confusion matrices obtained using baseline method (BoW+ *χ*^2^) and the proposed AMK type II kernel. When comparing the confusion matrices to the counterparts from KTH dataset, Figure 4(a) *vs.* Figure 5(a) and Figure 4(b) *vs.* Figure 5(b), it can be seen that the two baseline methods perform much worse on the Sneeze-Cough dataset than on the KTH dataset. This loss in accuracy suggests that the Sneeze-Cough dataset is much more challenging than the KTH dataset.

Meanwhile, the actions of *sneeze* and *cough* seems to be more correlated with the subset of actions {*call*, *drink*, *scratch* }, rather than the rest ones of {*stretch*, *wave*, *wipe* }. This might due to the fact that for the action subset {*sneeze*, *cough*, *call*, *drink*, *scratch* }, hands are usually placed near the face; While for {*stretch*, *wave*, *wipe* }, hands are often placed further away from the face. The gain in accuracy by adopting the AMK II kernel is also evident, when we compare the matrices (c) and (a) (or (d) and (b)) in Figure 5 for the KTH dataset, as well as by comparing the same two pair of matrices in Figure 4 for the Sneeze-Cough dataset. For KTH, an improvement of around five points has been observed for the averaged diagonal elements; As for Sneeze-Cough, this improvement is around ten points. These observations hold valid for both features.

Our major interest in this dataset is to recognize flu-like symptoms (sneeze and cough) from the background actions. Therefore, binary classification experiments are conducted to examine the performance of the proposed AMK kernels, and the results are listed in Table 2. For each AMK kernel type, in addition to the (precision, recall) pair, the accuracy measure provides an easy-to-compare summary of its performance. On average, we can see that this dataset is rather challenging. The best method merely reach an accuracy of around 44.4*%*, which can be partially explained by the large variations within cough/sneeze over various genders/ages/views, as well as their similar space-time appearances to those of the background actions. For both cuboid and HOGHOF local features, we see significant improvement in accuracy by using specific AMKs compared to baseline methods. Take cuboid feature for example, we observe that using the AMK type I kernel with *U*_*b*_ and *S*=27 leads to an increase of accuracy by 12.5 points. Interestingly, although the best results (*i.e.,* 43.4*%* vs. 44.4*%*) are similar for both local features, they are in fact obtained from different type of AMK kernels.

**Table 2:** **Comparisons of recognition accuracies on Sneeze-Cough dataset**

Brief description	Prec. %	Rec. %	Acc. %
cuboid + BoW + *χ* ^2^ (baseline)	49.5	45.0	30.9
cuboid + AMK I *U*_*c*_ S=6	69.5	47.5	39.3
cuboid + AMK I *U*_*b*_ S=27	78.7	47.2	43.4
cuboid + AMK I B S=2	50.0	54.3	35.2
cuboid + AMK II	55.3	62.1	41.3
HOGHOF + BoW + *χ* ^2^ (baseline)	52.6	50.0	34.4
HOGHOF + AMK I *U*_*c*_ S=3	63.7	48.3	37.9
HOGHOF + AMK I *U*_*b*_ S=27	69.7	40.8	34.6
HOGHOF + AMK I B S=5	60.8	49.2	37.3
HOGHOF + AMK II	58.9	64.4	44.4

Comparisons of recognition accuracies on Sneeze-Cough dataset. Here shorthands of AMK I and II are used for AMK type I and type II kernels, respectively. *U*_*c*_ (*U*_*b*_) refers to the concentric (block) partition scheme in unary extension. B is for binary extension. Note a *different* accuracy measure of  is used here.

## Conclusion

In this paper, we develop a new family of kernels in the proposed approach that explicitly integrates the two important aspects of action local features: space-time layout and BoW representations. Meanwhile, a new public health action dataset is introduced in this paper, to facilitate the study of detecting typical flu-like symptoms in public areas. This dataset is shown to be significantly different from and is more challenging than established datasets such as the KTH dataset. We demonstrate that our approach, while achieving competitive performance on the well-studied KTH dataset, produces reasonable results for this unique and challenging sneeze-cough dataset.

For ongoing work, we would extend the current approach to retrieve flu-like behavior symptoms from video archives, which often contain multiple persons simultaneously performing a series of actions, often in crowded environments. In particular, we plan to work with real surveillance datasets and test correlation of daily or weekly average sneeze/cough incidence with public health records of respiratory disease trends over time to show utility of the approach and if it is following or preceding reported peaks from hospital or doctor visit-based reporting systems. We envision that this approach can also be useful for detection of a variety of emergency situations triggering respiratory symptoms such as fires, gas leaks or chemical spills from accidents or even terrorist attacks.

## Endnotes

^1^ The dataset is made available at http://web.bii.a-star.edu.sg/~chengli/FluRecognition.htm.

^2^ TP, TN, FP, and FN refer to True Positive, True Negative, False Positive and False Negative, respectively.
